# RNA-Seq Reveals the Role of miR-29c in Regulating Inflammation and Oxidative Stress of Bovine Mammary Epithelial Cells

**DOI:** 10.3389/fvets.2022.865415

**Published:** 2022-04-01

**Authors:** Jian Yang, Qi-Chao Hu, Jin-Peng Wang, Qian-Qian Ren, Xing-Ping Wang, Zhuo-Ma Luoreng, Da-Wei Wei, Yun Ma

**Affiliations:** ^1^School of Agriculture, Ningxia University, Yinchuan, China; ^2^Key Laboratory of Ruminant Molecular Cell Breeding, Ningxia Hui Autonomous Region, Yinchuan, China

**Keywords:** dairy cow, mastitis, immune, oxidative stress, RNA-seq

## Abstract

Healthy mammary gland is essential for milk performance in dairy cows. MicroRNAs (miRNAs) are the key molecules to regulate the steady state of mammary gland in dairy cows. This study investigated the potential role of miR-29c in bovine mammary epithelial cells (bMECs). RNA sequencing (RNA-seq) was used to measure the transcriptome profile of bovine mammary epithelial cells line (MAC-T) transfected with miR-29c inhibitor or negative control (NC) inhibitor, and then differentially expressed genes (DEGs) were screened. The results showed that a total of 42 up-regulated and 27 down-regulated genes were found in the miR-29c inhibitor group compared with the NC inhibitor group. The functional enrichment of the above DEGs indicates that miR-29c is a potential regulator of oxidative stress and inflammatory response in bMECs through multiple genes, such as forkhead box O1 (*FOXO1*), tumor necrosis factor-alpha (*TNF-*α), and major histocompatibility complex, class II, DQ alpha 5 (*BoLA-DQA5*) in the various biological process and signaling pathways of stress-activated mitogen-activated protein kinase (MAPK) cascade, Epstein-Barr virus infection, inflammatory bowel disease, etc. The results imply that miR-29c plays an important role in a steady state of bMECs or cow mammary gland and may be a potential therapeutic target for mastitis in dairy cows.

## Introduction

The bovine mammary epithelial cells (bMECs) are the most important cell group of mammary tissues, which act in milk synthesis and innate immunity (1). When the cow mammary gland is exposed to exogenous pathogenic bacteria, bMECs are stimulated to produce reactive oxygen species (ROS), nitric oxide (NO), and pro-inflammatory cytokines, thereby initiating oxidative stress and inflammatory response (2, 3). In the process of dairy farming, the high incidence of mastitis affects the health and economic benefits of dairy cows (4).

MicroRNAs (miRNAs) are small non-coding RNA with a length of about 22 nt that can inhibit the expression levels of genes by targeting the 3' untranslated region (3' UTR) of the messenger RNAs (mRNAs), thereby regulating the biological processes of humans and other animals (5, 6). Previous studies observed that miRNAs could regulate the immunity, proliferation and apoptosis of bMECs. miR-21-3p promotes triglyceride synthesis of bMECs through direct and targeted regulation of elongation-of-very-long-chain (ELOVL) fatty acid elongase 5 (*ELOVL5*) expression (7). miR-24-3p targets multiple endocrine neoplasia type 1 (*MEN1*)/*menin* in bovine mammary epithelial cells line (MAC-T) and regulates cell proliferation and milk protein synthesis in the form of negative feedback (8). miR-146a targets the toll-like receptor 4 (TLR4)/tumor necrosis factor receptor (TNFR)-associated factor 6 (TRAF6)/nuclear factor kappa B (NF-κB) pathway and regulates lipopolysaccharide (LPS)-induced inflammatory response in bMECs by a negative feedback mechanism (9). miR-29 family regulates the synthesis and secretion of milk components, cell proliferation, and apoptosis by targeting DNA-methyltransferase 3A/3B *(DNMT-3A/-3B*) in bMECs (10). In mice and humans, the miR-29 family is a series of small RNA molecules closely related to inflammation or apoptosis (11–13), and miR-29c can target leukemia inhibitory factor (*LIF*) in primary intestinal epithelial cells (ICEs) and is involved in the regulation of proliferation, apoptosis, and immune function, thereby regulating Ulcerative colitis (UC) (14). Previous study found that the expression of miR-29c was down-regulated in mammary tissues samples of cows with *Escherichia coli*-induced mastitis (15) and cows with clinical mastitis (16). However, there is no report on the involvement of miR-29c in bMECs immune response regulation.

In this study, RNA sequencing (RNA-seq), Gene Ontology (GO) and Kyoto Encyclopedia of Genes and Genomes (KEGG) annotations were used to analyze the effects of miR-29c inhibition on the transcriptome profile of bMECs to explore the role of miR-29c in bMECs.

## Materials and Methods

### Cell Culture and Identification

The previously stored MAC-T cells (17) in liquid nitrogen were resuscitated and cultured with 10 mL Dulbecco's Modified Eagle Medium/Nutrient Mixture F-12 (DMEM/F12) medium (Hyclone, UT, USA) containing 10% fetal bovine serum (System Biosciences, Mountain View, CA, USA) in a 5% CO_2_ humidified incubator at 37°C and then passaged every 2 days.

MAC-T cells were identified by immunofluorescence analysis with epithelial marker cytokeratin 18 (18). The cells were inoculated in a six-well plate (NEST, Wuxi, Jiangsu, China). When the cell confluence was 80%, the medium was discarded. The cells were rinsed three times with PBS, and fixed at room temperature for 20 min with pre-cooled 4% paraformaldehyde (Sigma-Aldrich, St. Louis, MO, USA), then added 0.5% Triton X-100 (Sigma-Aldrich, St. Louis, MO, USA), and incubated at room temperature for 5 min. After blocking for 1 h at room temperature in 2% BSA. the cells were incubated with primary antibody for cytokeratin 18 (1:500, Santa Cruz, Dallas, TX, USA) overnight at 4°C. The cells was incubated with Cy3-labeled goat anti-mouse IgG (1:200, Beyotime, Shanghai, China) for 1 h at room temperature, then incubated with DAPI (Beyotime, Shanghai, China) for 10 min at room temperature, followed by seal the coverslip and imaging under fluorescence inverted microscope (Olympus, Shinjuku-ku, Tokyo, Japan).

### Cell Transfection

The miR-29c inhibitor and NC (negative control) inhibitor were designed and synthesized by Guangzhou RiboBio Co., Ltd. (Guangzhou, China). MAC-T cells were inoculated into a six-well cell culture plate (NEST, Wuxi, Jiangsu, China), a fresh medium was added before transfection when the cell confluence was 60~70%. X-treme GENE™ HP DNA Transfection Reagent (Roche, Basel, Switzerland) was used for instantaneous transfection of miR-29c inhibitor (100 nM) or NC inhibitor (100 nM) according to the manufacturer's instructions. The transfection efficiency was observed under fluorescence inverted microscope (Olympus, Shinjuku-ku, Tokyo, Japan) and detected by quantitative real-time polymerase chain reaction (qRT-PCR) of miR-29c in the transfected cells. The MAC-T cells were harvested at 48 h after transfection for RNA extraction.

### Construction of Transcriptome Libraries and Sequencing

Total RNA of 6 MAC-T cells samples, including 3 replicates of miR-29c inhibitor transfected cells and 3 replicates of NC inhibitor transfected cells, were extracted by TRIzol reagent (Takara, Beijing, China) for constructing transcriptome libraries. The integrity of the RNA samples were detected by agarose gel electrophoresis, and the purity was detected by Nanodrop (Thermo Fisher Scientific, Waltham, MA, USA). The optical density (*OD*_260_/*OD*_280_) of all RNA samples ranged from 1.8 to 2.0, indicating that the RNA samples were qualified. Then, 1 μg total RNA was used to construct transcriptome library by ABclonal mRNA-seq Lib Prep Kit (ABclonal, Wuhan, Hubei, China), and mRNA was enriched with magnetic beads with oligo (dT). mRNA was broken into short fragments by fragmentation buffer and used as a template. First-strand of complementary DNA (cDNA) was synthesized by random hexamers, and then the second-strand of cDNA was synthesized by adding buffer, deoxynucleotide triphosphate (dNTPs), DNA polymerase I, and RNase H. Then, AMPure XP beads were used to purify double-stranded cDNA. Purified double-stranded cDNA ends were repaired. A-tails were added, and sequencing adapters were connected. Then, AMPure XP beads were used to select segment sizes. Polymerase chain reaction (PCR) amplification was performed, and AMPure XP beads were used to purify the PCR products to get the final library. After the library's construction, the insert size and effective concentration of the library were detected to ensure the quality of the library. After the quality inspection, the 6 libraries were sequenced on Illumina Novaseq 6000 (Illumina, Inc., San Diego, CA, USA) high-throughput platform in shanghai applied protein technology co. ltd. (Shanghai, China).

### Quality Control and Mapping of Sequencing Data

After high-throughput sequencing, raw data were stored in FASTQ file format. Clean reads were obtained after the quality control of raw reads by Perl, such as removal of adapters sequence, filtering out low-quality reads and filtering out reads with N ratio >5%. Clean reads were compared to mapped reads in the *Bos taurus* reference genome UMD3.1 (http://oct2018.archive.ensembl.org/Bos_taurus/Info/Index) using HiSAT2 software (http://daehwankimlab.github.io/hisat2/).

### Differential Expression Analysis of Genes

The expression level of each gene in each sample was calculated using FeatureCounts software (http://subread.sourceforge.net/). The gene expression level was expressed as FPKM (number of bases per million compared segments to transcript per thousand) (19). DESeq2 (http://bioconductor.org/packages/release/bioc/html/DESeq2.html) was used to compare the genes expression levels of miR-29c inhibitor and NC-inhibitor groups, with *P* < 0.05 & | log${}_{2}^{\text{foldchange}}$ | > 1 as the standard significance of the difference between the groups.

### Reverse Transcription and qRT-PCR

The reverse transcription reactions of miR-29c and mRNAs were carried out by stem-loop structure primer (20) (Supplementary Table S1) and the mixture of Oligo (dT) primers and random 6 mers, respectively. All reverse transcription reactions were performed using 1 μg total RNA as the template and using PrimeScript™ RT Reagent Kit with gDNA Eraser (Takara Biomedical Technology Co., Ltd., Beijing, China) according to the manufacturer's instructions.

qRT-PCR was used to detect the transfection efficiency of miR-29c and validate the differentially expressed genes (DEGs) obtained by RNA-seq. qRT-PCR was performed using 2× M5 HiPer SYBR Premix EsTaq plus kit (Mei5 Biotechnology Co., Ltd., Beijing, China) on a CFX96 assay system (Bio-Rad Laboratories, Inc., CA, USA) in a 20 μL reaction system, including 10 μL 2 × M5 HiPer SYBR Premix Es Taq (with Til RNaseH), 0.8 μL 10 μmol/L of each of forward and reverse primers, 2.0 μL cDNA (100 ng/μL) and 6.4 μL RNase-free ddH_2_O. The amplification conditions were as follows: pre-denaturation at 95°C for 30 s, followed by 40 cycles of denaturation at 95°C for 5 s and annealing/extension at 60°C for 30 s. The primers designed with Primer Premier 5 (PREMIER Biosoft International, Palo Alto, CA, USA) are presented in Supplementary Table S1.

The relative expression levels of miR-29c and DEGs were normalized by glyceraldehyde 3-phosphate dehydrogenase (*GAPDH*) and ribosomal protein S18 (*RPS18*) (21), and the data were calculated using the 2^−Δ*ΔCT*^ method (22). A significant difference was performed using SPSS 25.0 Student's *t*-tests to determine the statistical significance of the two groups, and *P* < 0.05 indicated significant difference, and *P* < 0.01 indicated extremely significant difference. All data were expressed as mean ± standard error of the mean (SEM).

### Functional Annotation of DEGs

The R package of clusterProfiler (version: 4.0.5) (23) was used for GO and KEGG enrichment analysis of DEGs, and terms with a *P* < 0.05 were considered significantly enriched.

## Results

### Detection of miR-29c Silencing Effect in MAC-T Cells

The identification of MAC-T cells was performed by detecting the expression of epithelial cell-specific cytokeratin 18. The results showed that cytokeratin 18 was expressed in all cells (Supplementary Figure S1), suggested that the MAC-T cells used in this study were reliable.

**Figure 1:**
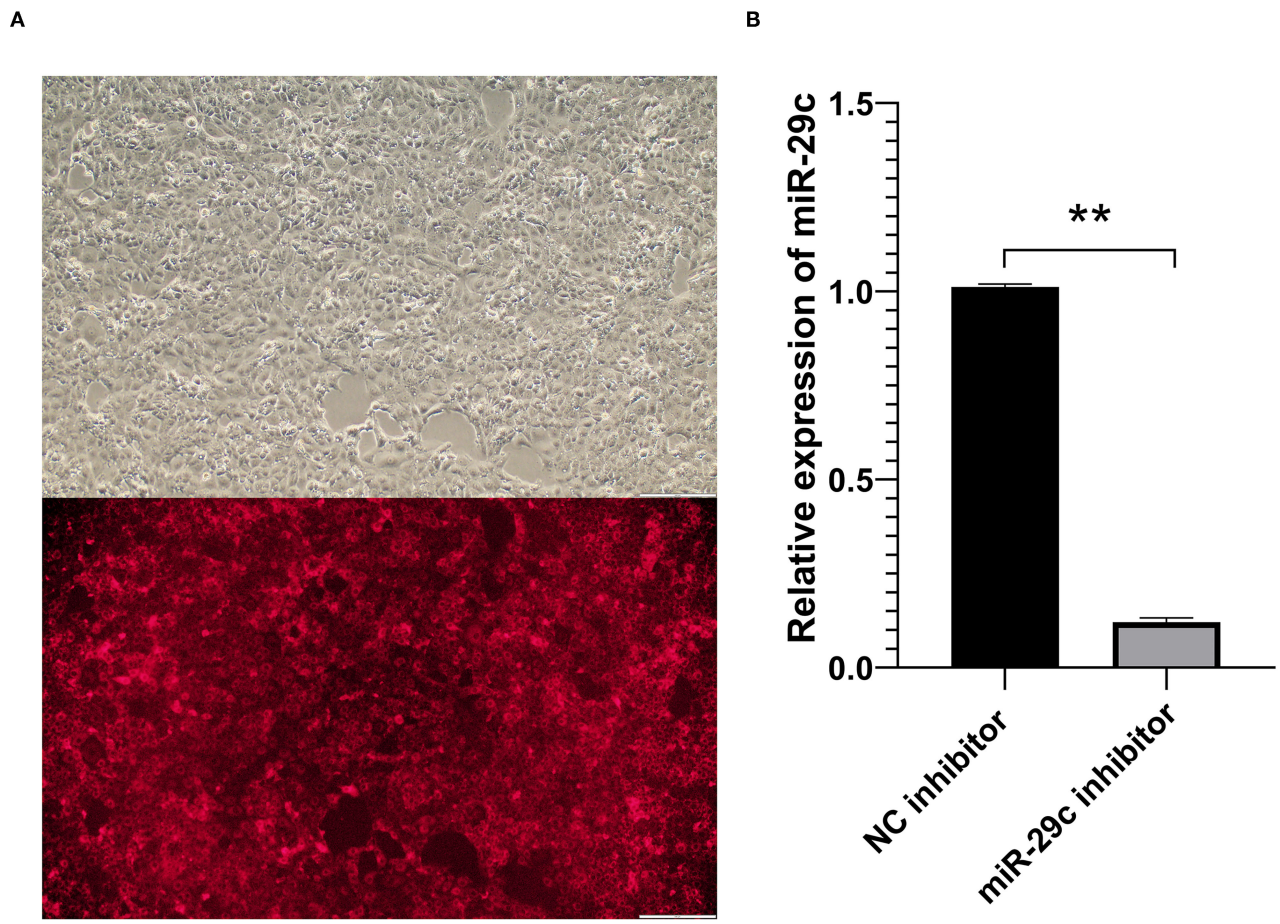
Transfection efficiency detection of bovine miR-29c in MAC-T cells. **(A)** Transfection efficiency of NC inhibitor in MAC-T cells observed by fluorescence microscopy. **(B)** The transfection efficiency of bovine miR-29c inhibitor in MAC-T cells were measured by qRT-PCR. Data are presented as mean ± SEM. **P < 0.01.

After MAC-T cells were transfected with a Cy3-labeled NC inhibitor and incubated for 24 h, red fluorescence in MAC-T cells were clearly observed (Figure 1A). The qRT-PCR results showed that the expression of miR-29c was significantly down-regulated in miR-29c inhibitor group compared with the NC inhibitor group (*P* < 0.01, Figure 1B). The above results indicated that the inhibitor successfully inhibited the expression of miR-29c in MAC-T cells.

### RNA-Seq Summary Statistics

**Figure 2:**
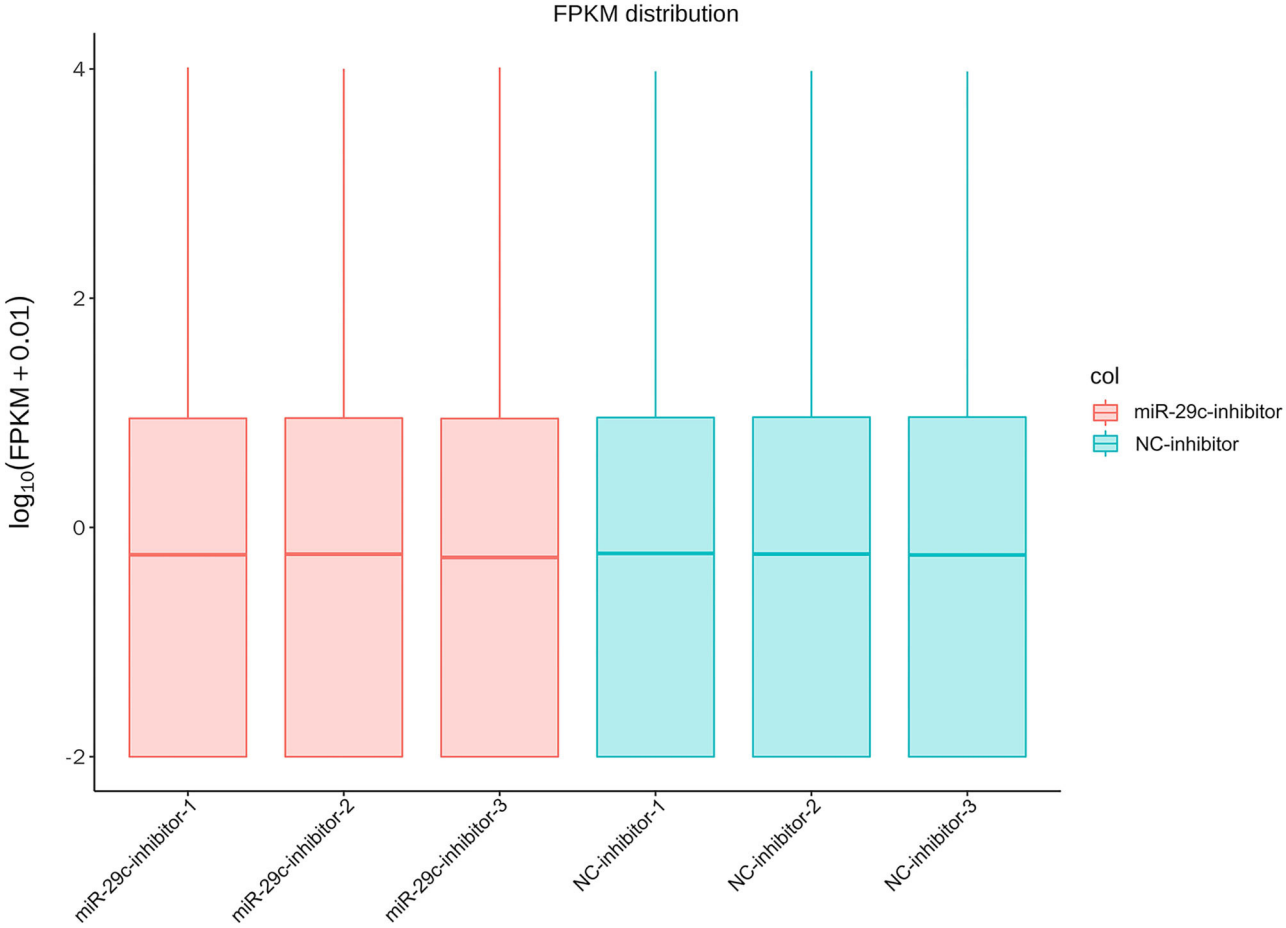
The boxplot of expression abundance (FPKM) of genes in each sample.

**Figure 3:**
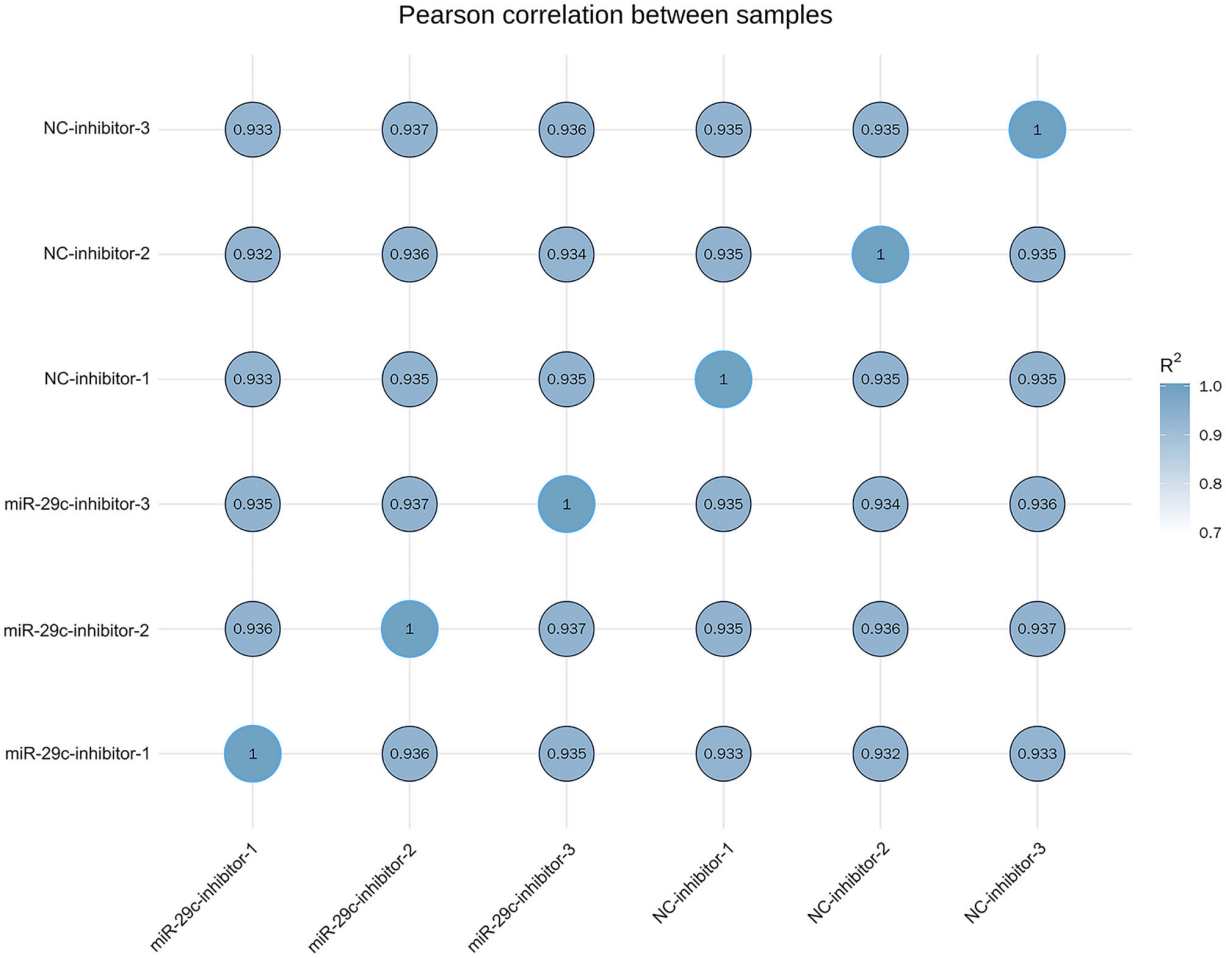
Heat map of the correlation coefficient between samples.

Total RNA was extracted from each sample of NC inhibitor and miR-29c inhibitor groups. Transcriptome libraries were established, and RNA-seq was performed. A total of 267 million clean reads were obtained by filtering the original data, and at least 5.67 G of the clean base was obtained from each sample, which could be further used for subsequent analysis. The Q30 of all samples were >92.6%, and the GC content ranged from 47.05 to 47.57% (Table 1). Clean reads were mapped to the bovine reference genome, and the total mapping rate reached 95.67–96.14%, among which 93.23–93.74% of clean reads had unique mapped positions on the reference sequence (Table 2). The boxplot of expression abundance (FPKM) of genes in each sample showed the overall gene expression abundance of different samples (Figure 2). Correlation analysis between samples showed that *R*^2^-values were >0.8 for each group of biological replicates (Figure 3). The above results indicated that our sequencing data met the requirements for the analysis of DEGs.

**Table 1:** Summary of RNA-seq data.

**Sample**	**Raw reads**	**Clean reads**	**Clean bases**	**Error (%)**	**Q20 (%)**	**Q30 (%)**	**GC (%)**
NC inhibitor-1	41433410	41141648	5.7G	0.03	97.59	93.02	47.07
NC inhibitor-2	46829314	46481256	6.42G	0.03	97.38	92.6	47.08
NC inhibitor-3	46103512	45797992	6.33G	0.03	97.69	93.28	47.05
miR-29c inhibitor-1	41238106	40934936	5.67G	0.03	97.43	92.73	47.29
miR-29c inhibitor-2	44529582	44186794	6.12G	0.03	97.5	92.89	47.34
miR-29c inhibitor-3	48910762	48539344	6.72G	0.03	97.7	93.33	47.57

**Table 2:** Overview of reads mapped to reference genome.

**Sample**	**Clean reads**	**Total mapped**	**Multiple mapped**	**Unique mapped**	**Non-splice reads**	**Splice reads**
NC inhibitor-1	41141648	39472120 (95.94%)	974286 (2.37%)	38497834 (93.57%)	24104014 (58.59%)	14393820 (34.99%)
NC inhibitor-2	46481256	44516188 (95.77%)	1120799 (2.41%)	43395389 (93.36%)	27311222 (58.76%)	16084167 (34.60%)
NC inhibitor-3	45797992	44030956 (96.14%)	1099954 (2.40%)	42931002 (93.74%)	26878587 (58.69%)	16052415 (35.05%)
miR-29c inhibitor-1	40934936	39185061 (95.73%)	984702 (2.41%)	38200359 (93.32%)	23783716 (58.10%)	14416643 (35.22%)
miR-29c inhibitor-2	44186794	42275231 (95.67%)	1077856 (2.44%)	41197375 (93.23%)	25608388 (57.95%)	15588987 (35.28%)
miR-29c inhibitor-3	48539344	46546820 (95.90%)	1216390 (2.51%)	45330430 (93.39%)	28118316 (57.93%)	17212114 (35.46%)

### Differential Expression Analysis of Genes

**Figure 4:**
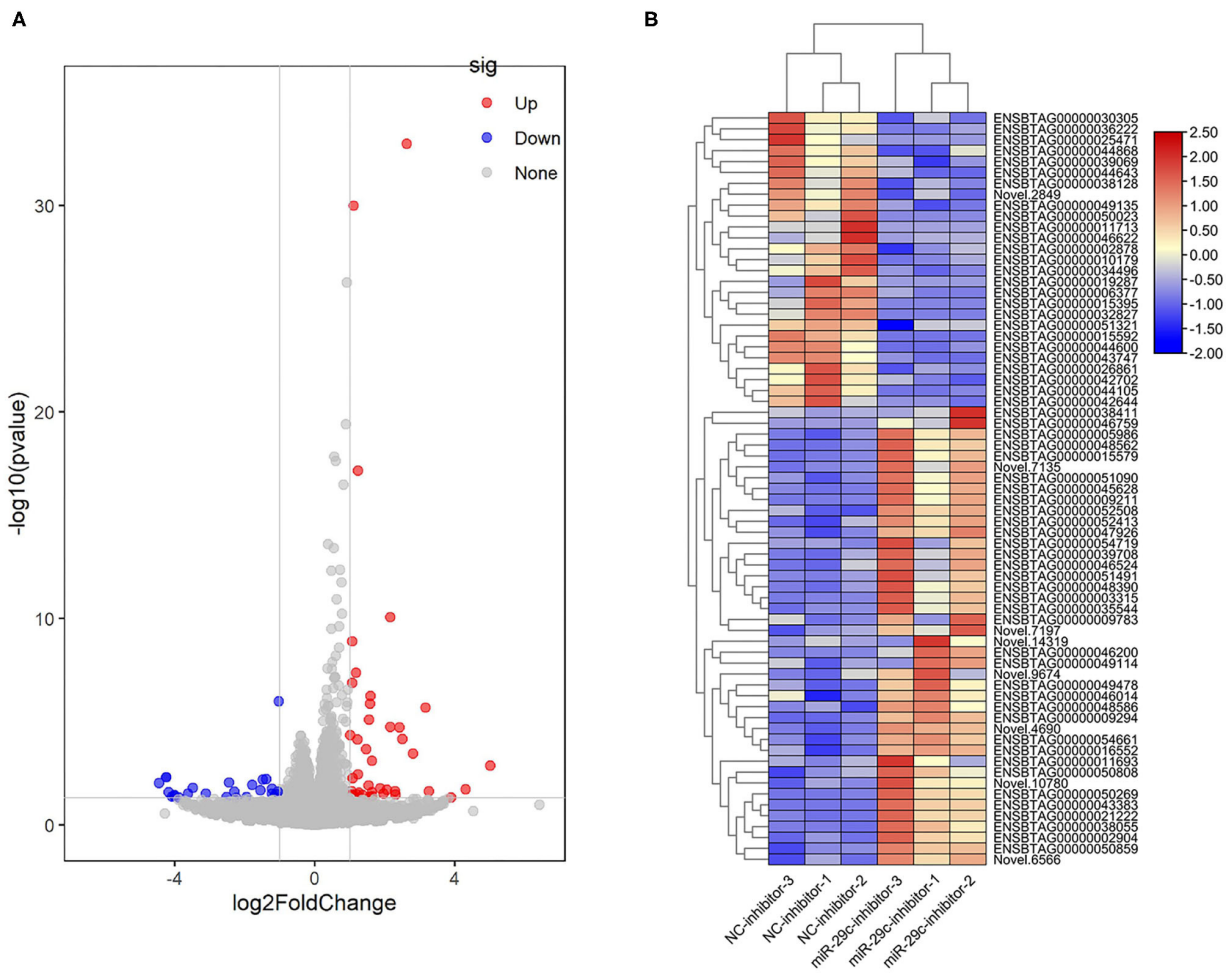
Analysis of DEGs in miR-29c inhibitor and NC inhibitor groups. **(A)** Volcano map of genes expression between the miR-29c inhibitor and NC inhibitor groups. The red points represent up-regulated genes, and the blue points represent down-regulated genes with statistical significance. **(B)** Cluster heat map of DEGs between the miR-29c inhibitor and NC inhibitor groups.

With *P* < 0.05 and |log${}_{2}^{\text{foldchange}}$|>1, a total of 42 genes were significantly up-regulated and 27 genes were significantly down-regulated in the miR-29c inhibitor group compared with the NC inhibitor group (Figures 4A,B, Supplementary Table S2). Three immune-related DEGs, namely forkhead box O1 (*FOXO1*), tumor necrosis factor-alpha (*TNF-*α), and major histocompatibility complex, class II, DQ alpha 5 (*BoLA-DQA5*) (24–26), were significantly down-regulated in the miR-29c inhibitor group (the values of log${}_{2}^{\text{foldchange}}$ of *FOXO1, TNF-*α, and *BoLA-DQA5* were −1.0581, −4.1784, and −1.394, respectively). The above results suggested that miR-29c might regulate the immune response in MAC-T by regulating these genes.

### Validation of RNA-Seq Results by qRT-PCR

**Figure 5:**
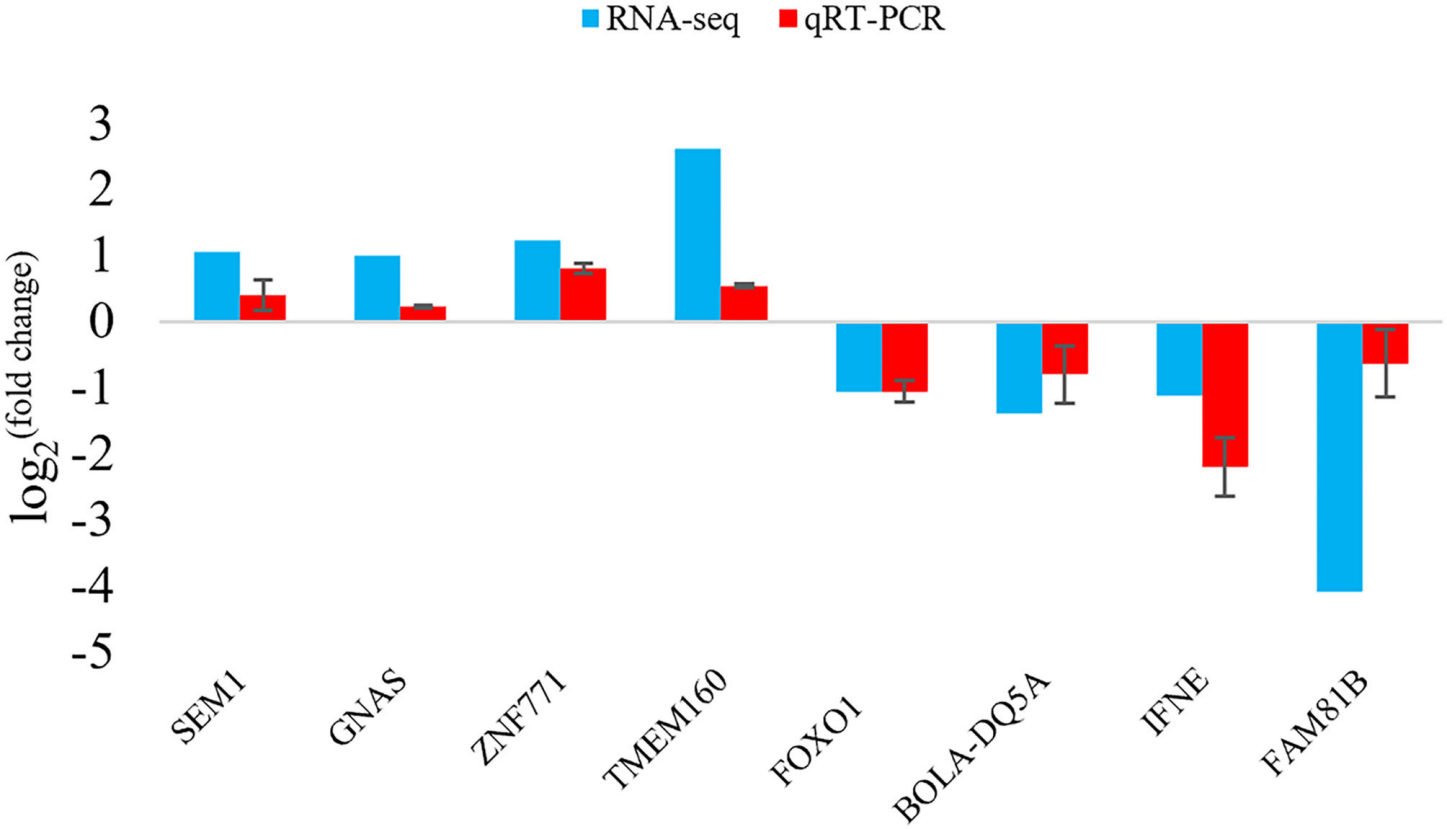
qRT-PCR validation of DEGs.

To verify the results of the deep sequencing and the DEGs obtained by RNA-seq analysis, a total of 8 DEGs were randomly selected for qRT-PCR. Comparing the qRT-PCR results with the RNA-seq results, the expression trends of 8 DEGs obtained by two methods were similar (Figure 5), indicating that the results of RNA-seq and the screening of DEGs were reliable.

### GO and KEGG Analysis of DEGs

**Figure 6:**
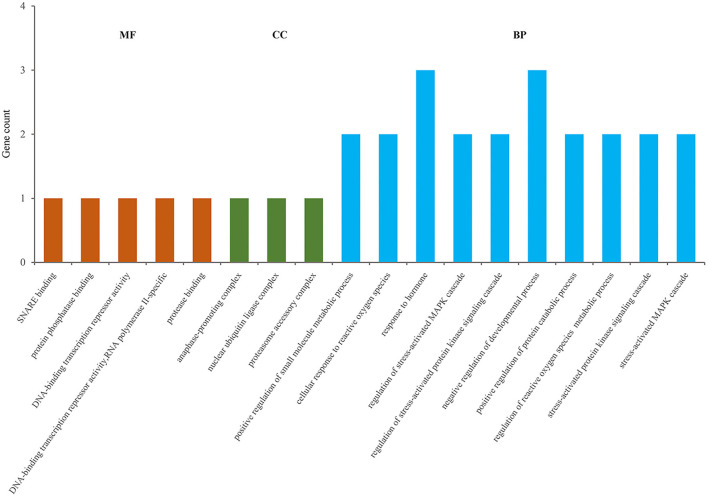
GO annotation of DEGs, and the significance level of enrichment was set at *P* < 0.05 (top10).

**Figure 7:**
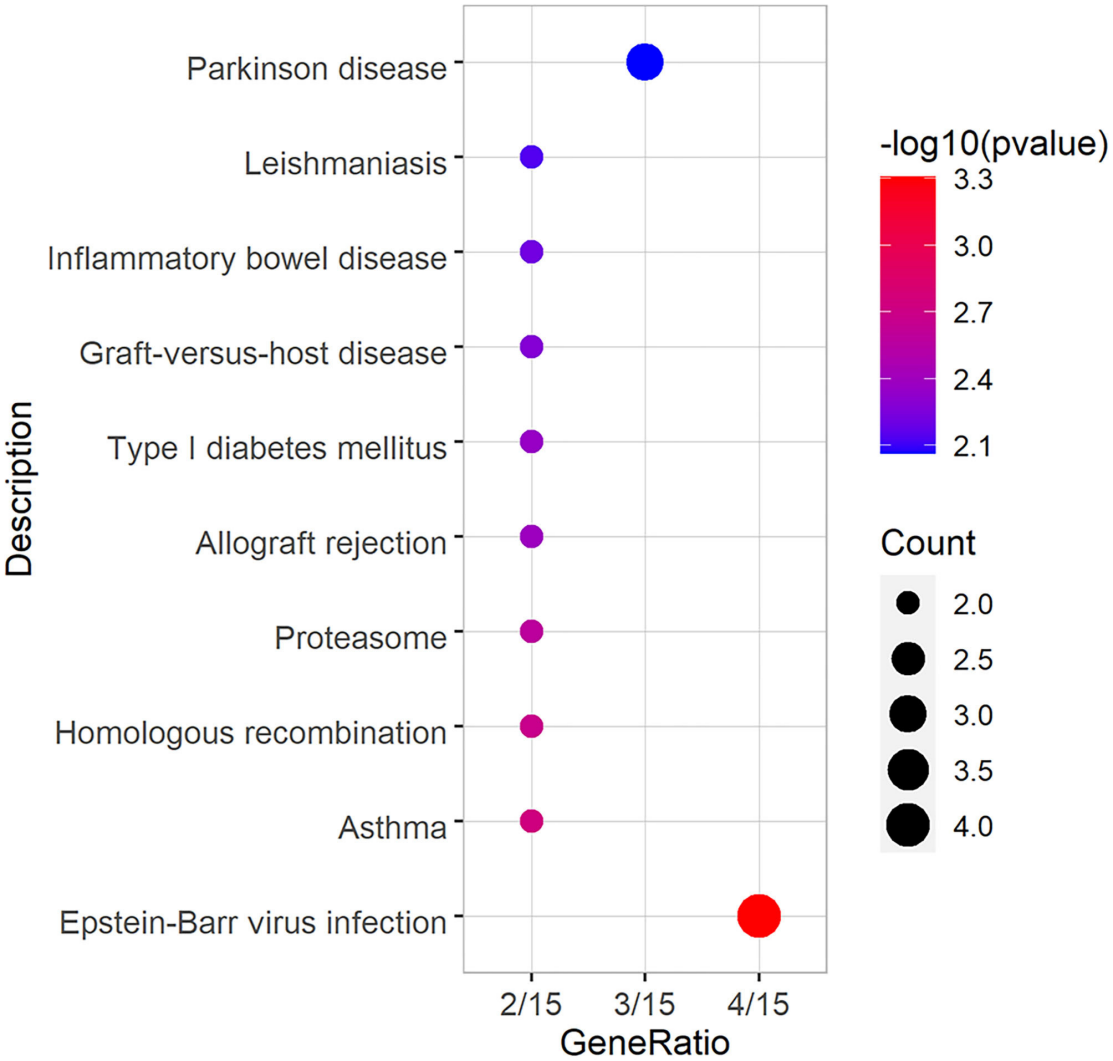
KEGG annotation of DEGs, and the significance level of enrichment was set at *P* < 0.05 (top10).

Total DEGs were annotated to cellular components (CC), molecular function (MF), and biological process (BP) through GO enrichment analysis. Figure 6, Supplementary Table S3 shows the top 10 entries in each group. The results showed that DEGs were significantly enriched in CC entries, such as anaphase-promoting complex, nuclear ubiquitin ligase complex, and proteasome accessory complex. MF annotation indicated that the DEGs might play a role in SNARE binding, protein phosphatase binding and protease binding. BP annotation showed that the DEGs might be involved in regulating stress-activated mitogen-activated protein kinase (MAPK) cascade, stress-activated protein kinase signaling cascade, and cellular response to ROS. KEGG enrichment was used to analyze the possible key signaling pathways involved in DEGs (Figure 7, Supplementary Table S4). DEGs were significantly enriched in immune-related signaling pathways, such as asthma, allograft rejection, Epstein-Barr virus infection, and inflammatory bowel disease. The analysis showed that most of these results were related to *FOXO1, TNF-*α, and *BOLA-DQA5*, which were significantly down-regulated in the miR-29c inhibitor group and were previously tested to be involved in the regulation of oxidative stress and immune response. The above results suggested that miR-29c might be involved in the regulation of oxidative stress and the immune response of MAC-T through the above-mentioned genes.

## Discussion

When endogenous or exogenous ROS and stimulation factors, such as LPS, stimulate bMECs, the oxidative stress and inflammatory response generated by bMECs reduce vitality and affect the health of the mammary gland, resulting in lactation dysfunction, which is the main cause of cow mastitis (27–30). The occurrence of mastitis leads to extensive changes in transcriptome profiles, including miRNA. Some important differentially expressed miRNAs (DEmiRNAs) have been screened out in bovine mammary tissues infected with pathogenic bacteria or in LPS-induced bMECs, such as miR-145 (31), miR-146a (9), and miR-223 (32, 33), which affect the inflammatory response of bMECs by regulating the activation of immune-related signaling pathways, such as TLRs signaling pathway, NF-κB signaling pathway and Janus kinase/signal transducer and activator of transcription (JAK/STAT) signaling pathway (9, 34–36). In addition, the study have shown that miR-141 and miR-200a are closely related to the regulation of oxidative stress in bMECs (37).Thus, miR-29c, as a significant DEmiRNA related to cow mastitis, should determine its potential role in bMECs. In this study, we interfered with the expression of miR-29c in MAC-T cells using a specific inhibitor and detected its transcriptome changes relative to the NC inhibitor group by RNA-seq. A total of 69 DEGs were detected, and the result of qRT-PCR verified that result of RNA-seq were reliable. These DEGs are involved in immune-related biological processes and signaling pathways, such as stress-activated MAPK cascade, Epstein-Barr virus infection and inflammatory bowel disease signaling pathways. It is suggested that the inhibition of miR-29c can regulate the immune and stress responses of bMECs.

The MAPK cascade mainly consists of three kinases: MAPK kinase kinase, MAPK kinase, and MAPK, which are activated and phosphorylated downstream in turn and participate in regulating a variety of life processes, including cellular immunity, proliferation, and apoptosis (38–41). Similarly, oxidative stress is an important part of the cellular immune response (42, 43). Inhibition of the MAPK signaling pathway and the expression of genes related to oxidative stress can effectively reduce the inflammatory injury of bMECs induced by LPS (44, 45). Our results found that *FOXO1* and *TNF-*α were significantly enriched in the above biological processes in bMECs. *FOXO1* is an important regulator of the cellular oxidative stress response (46), it was reported that *FOXO1* was significantly up-regulated in H_2_O_2_-induced H9C2 cells, while inhibition of *FOXO1* could up-regulate superoxide dismutase (SOD) levels and down-regulate malondialdehyde (MDA) and lactate dehydrogenase (LDH) levels, thus reducing oxidative stress and cell apoptosis induced by H_2_O_2_ (47). Furthermore, inhibition expression of *FOXO1* in the RAW264.7 cell line resulted in the down-regulation of pro-inflammatory genes, such as interleukin 1-beta (*IL-1*β), interleukin 6 (*IL-6*), and monocyte chemoattractant protein-1 (*MCP-1*) (48). Previous reports found that knockdown of miR-29c in breast cancer cells inhibited *FOXO1* expression but promoted the proliferation, migration, and invasion of breast cancer cells (49). *TNF-*α is an important regulator of the immune response in mammals under physiological or pathological conditions and can regulate the signaling pathways related to immune cell proliferation and apoptosis (50, 51). The expression of the *TNF-*α gene is positively correlated with the severity of dairy cow mastitis. Studies have found that miR-142-5p regulates LPS-induced bMECs inflammation by targeting Bcl-2-associated athanogene (*BAG5*), and it is positively correlated with *TNF-*α, *IL-1*β, *IL-6*, and interleukin 8 (*IL-8*) (52). Therefore, *FOXO1* and *TNF-*α were significantly down-regulated in the miR-29c inhibitor group, suggesting that down-regulation of miR-29c may reduce inflammation and oxidative stress, which was of great significance to the maintenance of bMECs steady state.

As a cell surface protein, soluble major histocompatibility complex (MHC) class II molecules (e.g., *BoLA-DQA family)* play an important role in maintaining immune homeostasis with the intracellular transport process (53). In this study, *BoLA-DQA5* was significantly enriched in inflammatory and immune-related signal pathways, such as Epstein-Barr virus infection and inflammatory bowel disease. Hou et al. (54) reported that cow mastitis might be regulated by *BoLA-DQA2* splice variants. In addition, studies have shown that the copy number variation of *BoLA-DQA5* may be related to various infection-related phenotypes in Holstein cows (55). And in the inflammatory diseases of cows, the expression of the *BoLA-DQA5* gene was significantly down-regulated in subclinical endometritis samples of cows on the 7th day of the estrus cycle compared with that of healthy cows, which may be involved in regulating antigen processing and presentation pathway (56). However, the potential effect of *BoLA-DQA5* on bMECs immune response needs further study.

It is widely known that miRNAs don't encode proteins, but they can directly or indirectly regulate the expressions of many genes (57). In previous reports, miR-29c was proved to target specific protein-1 (*SP1*) (11) and nuclear factor of activated T cells 5 (*NFAT5*) (58) to inhibit the inflammatory response in Parkinson's disease. However, miR-29c promotes the inflammatory response by targeting *LIF* in UC (14). Interestingly, the pathway for the above diseases were also enriched in our KEGG annotated results. In addition, a study was shown that inhibition of expression of miR-29c can help to reduce inflammation caused by sepsis (59). The above studies have shown that the role of miR-29c is different in different inflammation, which further highlights the importance of studying its role in bMECs. In this study, we identified the effect of miR-29c inhibition on the gene expression of MAC-T cells from a global perspective using RNA-seq technology, and the results indicated that miR-29c may regulate inflammatory responses and oxidative stress in bMECs. Meanwhile, considered the similarities of genes among different species, the target gene of miR-29c in other species still needs to be verified in dairy cow. Consequently, we predicted the potential target genes of miR-29c using the TargetScan database (http://www.targetscan.org/vert_80/) (Supplementary Table S5), and compared them with DEGs. What's surprising to us, though, is the fact that the predicted target genes did not include in the list of the up-regulated genes (log${}_{2}^{\text{foldchange}}$ > 1) in the miR-29c inhibitor group, the reason of which is that, the predicted target genes of miR-29c might not be identified under the criteria of DEGs screening adopted in this study. As we know, the roles of miRNA are extensive, sensitive and rapid, and the inhibition of miRNAs on target genes may be more significant at the protein level, rather than mRNA level (60, 61). In addition, the expressions of target genes of miRNAs may be affected by negative feedback and cascade regulation (62). In short, this study provides valuable evidence for the potential role of miR-29c in regulating inflammation and oxidative stress in bMECs, but its specific molecular mechanism, including target gene identification, remains to be further studied.

## Conclusion

In conclusion, RNA-seq revealed that the inhibition of miR-29c in MAC-T cells could lead to the up-regulation of 42 genes and the down-regulation of 27 genes. The functional enrichment of the DEGs indicated that miR-29c might be a potential regulator of oxidative stress and inflammatory response in bMECs through multiple genes, such as *FOXO1, TNF-*α, and *BoLA-DQA5*, which enriched in stress-activated MAPK cascade, Epstein-Barr virus infection and inflammatory bowel disease signaling pathways. The above results imply that miR-29c plays an important role in the steady state of bMECs or cow mammary gland and may be a potential therapeutic target for mastitis in dairy cows.

## Data Availability Statement

The datasets presented in this study can be found in online repositories. The names of the repository/repositories and accession number(s) can be found in the article/Supplementary Material, and GEO DATABASE (https://www.ncbi.nlm.nih.gov/geo/query/acc.cgi?acc=GSE195508).

## Author Contributions

JY, X-PW, and Z-ML designed the experiments. JY and Q-CH completed the experiments. JY, X-PW, Z-ML, and J-PW analyzed the data. JY drafted the paper. J-PW, Q-QR, X-PW, Z-ML, YM, and D-WW revised the manuscript. All authors contributed to the article and approved the submitted version.

## Funding

This research was supported by grants from the National Natural Science Foundation of China (No. 31960652), the Key Research and Development Project (Talent Introduction Project) of Ningxia Hui Autonomous Region (No. 2019BEB04002), and the Introducing Talent Research Project of Ningxia University (No. 030900001926).

## Conflict of Interest

The authors declare that the research was conducted in the absence of any commercial or financial relationships that could be construed as a potential conflict of interest.

## Publisher's Note

All claims expressed in this article are solely those of the authors and do not necessarily represent those of their affiliated organizations, or those of the publisher, the editors and the reviewers. Any product that may be evaluated in this article, or claim that may be made by its manufacturer, is not guaranteed or endorsed by the publisher.

## Supplementary Material

Supplementary Figure S1 Identification of MAC-T cells by immunofluorescence with cytokeratin 18.

Supplementary Table S1 The list of all primer sequences for qRT-PCR.

Supplementary Table S2 The list of DEGs in miR-29c inhibitor group vs. NC inhibitor group.

Supplementary Table S3 The list of GO enrichment analysis results for DEGs.

Supplementary Table S4 The list of KEGG enrichment analysis results for DEGs.

Supplementary Table S5 The list of the predicted target genes for miR-29c.
