# Supplementary material for: RNA-Seq Reveals the Role of miR-29c in Regulating Inflammation and Oxidative Stress of Bovine Mammary Epithelial Cells
Source: Front Vet Sci. 2022 Apr 1;9:865415. doi: 10.3389/fvets.2022.865415 (PMC9011060; doi:10.3389/fvets.2022.865415)
Supplement: Supplementary Figure S1 — Identification of MAC-T cells by immunofluorescence with cytokeratin 18. [file Data_Sheet_1.ZIP › Figure S1. Identification of MAC-T cells by immunofluorescence with cytokeratin 18.pdf]

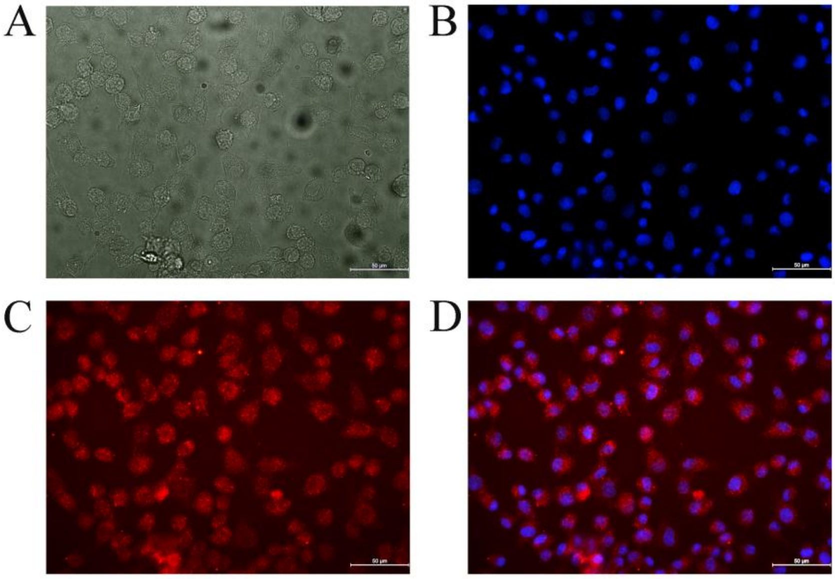

Figure S1. Identification of MAC-T cells by immunofluorescence with cytokeratin 18 (200×). (A) Bright field. (B) DAPI staining. (C) Immunofluorescence with cytokeratin 18 labeled with Cy3. (D) Merge of B and C. The slightly rounded cell morphology may be due to the instability of the cell attachment culture dish during immunofluorescence staining.
